# Nutraceuticals and Herbs in Reducing the Risk and Improving the Treatment of COVID-19 by Targeting SARS-CoV-2

**DOI:** 10.3390/biomedicines9091266

**Published:** 2021-09-18

**Authors:** Priti Tagde, Sandeep Tagde, Pooja Tagde, Tanima Bhattacharya, Shams Minhaz Monzur, Md. Habibur Rahman, Pavel Otrisal, Tapan Behl, Syed Shams ul Hassan, Mohamed M. Abdel-Daim, Lotfi Aleya, Simona Bungau

**Affiliations:** 1 Bhabha Pharmacy Research Institute, Bhabha University, Bhopal 462026, India; 2 PRISAL Foundation, Pharmaceutical Royal International Society, Bhopal 462042, India; sandeeptagde91@gmail.com; 3 Practice of Medicine Department, Government Homeopathic Medical College, Bhopal 462003, India; tagde_pooja@rediffmail.com; 4 School of Chemistry and Chemical Engineering, Hubei University, Hubei 430062, China; btanima1987@gmail.com; 5 Techno India NJR Institute of Technology, Udaipur 313003, India; 6 Integro Global Ltd., Dhaka 1206, Bangladesh; minhazgq@gmail.com; 7 Department of Pharmacy, Jagannath University, Sadarghat, Dhaka 1100, Bangladesh; 8 Department of Pharmacy, Southeast University, Banani, Dhaka 1213, Bangladesh; 9 Faculty of Physical Culture, Palacký University Olomouc, 77111 Olomouc, Czech Republic; pavel.otrisal@upol.cz; 10 Department of Pharmacology, Chitkara College of Pharmacy, Chitkara University, Punjab 140401, India; tapanbehl31@gmail.com; 11 Shanghai Key Laboratory for Molecular Engineering of Chiral Drugs, School of Pharmacy, Shanghai Jiao Tong University, Shanghai 200240, China; Shams1327@yahoo.com; 12 Department of Natural Product Chemistry, School of Pharmacy, Shanghai Jiao Tong University, Shanghai 200240, China; 13 Department of Pharmaceutical Sciences, Batterjee Medical College, P.O. Box 6231, Jedah 21442, Saudi Arabia; abdeldaim.m@vet.suez.edu.eg; 14 Pharmacology Department, Faculty of Veterinary Medicine, Suez Canal University, Ismailia 41522, Egypt; 15 Chrono-Environment CNRS 6249, Université de Franche-Comté, 25000 Besançon, France; lot-fi.aleya@univ-fcomte.fr; 16 Department of Pharmacy, Faculty of Medicine and Pharmacy, University of Oradea, 410028 Oradea, Romania; 17 Doctoral School of Biological and Biomedical Sciences, University of Oradea, 410087 Oradea, Romania

**Keywords:** COVID-19, SARS-CoV-2, probiotics, herbs, nutraceuticals, supplements, therapy

## Abstract

The worldwide transmission of acute respiratory syndrome coronavirus 2 (SARS-CoV-2) as a deadly or devastating disease is known to affect thousands of people every day, many of them dying all over the planet. The main reason for the massive effect of COVID-19 on society is its unpredictable spread, which does not allow for proper planning or management of this disease. Antibiotics, antivirals, and other prescription drugs, necessary and used in therapy, obviously have side effects (minor or significant) on the affected person, there are still not clear enough studies to elucidate their combined effect in this specific treatment, and existing protocols are sometimes unclear and uncertain. In contrast, it has been found that nutraceuticals, supplements, and various herbs can be effective in reducing the chances of SARS-CoV-2 infection, but also in alleviating COVID-19 symptoms. However, not enough specific details are yet available, and precise scientific studies to validate the approved benefits of natural food additives, probiotics, herbs, and nutraceuticals will need to be standardized according to current regulations. These alternative treatments may not have a direct effect on the virus or reduce the risk of infection with it, but these products certainly stimulate the human immune system so that the body is better prepared to fight the disease. This paper aims at a specialized literary foray precisely in the field of these “cures” that can provide real revelations in the therapy of coronavirus infection

## 1. Introduction

Wuhan (P.R. of China) was the first city where the coronavirus was identified in December 2019 [1]. It produces a severe acute respiratory disease that has spread dramatically throughout the world, and the World Health Organization (WHO) announced a pandemic on 11 March 2020 [2]. The virus belongs to a large family named *Coronaviridae*, subfamily *Coronavirinae*, that affects animals and mammals [3]. The highest numbers of cases were reported from the United States of America, India, Brazil, Russia, France, United Kingdom, Turkey, with many infected persons and deaths varying daily by continents, regions, and countries [4].

This virus is transferred through aerosol drops [5] affecting the nose, mouth, and eyes of an infected person. Common COVID-19 clinical signs include fever, exhaustion, dry cough, flu, dyspnea, headache, sore throat, etc., its essential manifestation of infection being pneumonia. The SARS-CoV-2 medical distal continuum can range from asymptomatic patients to critical pneumonia resulting in acute respiratory distress syndrome sometimes contributing to multi-organ failure (MOF), and this infectious disease continues evolving and growing worldwide [6]. Some patients are characterized mainly by dyspnea and hypoxemia, which can gradually evolve into a moderate respiratory condition requiring oxygen treatment.

On the other hand, septic trauma, metabolic acidosis, coagulation dysfunction with disseminated intravascular coagulation (DIC), and multiple organ dysfunction syndromes (MODS) often occur in other patients that develop rapidly into acute respiratory distress syndrome (ARDS) [7]. COVID-19 has thus emerged as a flexible, multi-system, multi-organ disarray that causes pathogenic effects at the level of multiple organs via a fairly omnipresent target [8]. All age ranges are subject to the virus, and elderly patients with co-occurring diseases are more likely to experience a severe illness. Scientists have tried hard to produce an affordable and efficient vaccine with high therapeutic value, but few vaccines are already available to date; additionally, in seeing such a high rise in the daily cases of COVID-19, it would be necessary to implement precautions and hygienic measures to monitor and reduce human-to-human transmission of SARS-CoV-2 before there is any successful intervention or consumption of dietary supplements along with herbal remedies which seem to be a very effective way to stop or at least to reduce this pandemic [9]. Most researchers’ efforts are focused on recognizing and understanding the pathogenesis of COVID-19 [10]. If this virus’s characteristics and the mechanism of action of this virus causing the pandemic can be understood [11], evaluated, and established, an effective treatment protocol and specific logical therapy could be easily identified.

Although there is no proof that any antiviral medication demonstrates a 100% successful outcome, remdesivir has been identified as one of the drugs which is successful in shortening the hospitalization period of patients infected with COVID-19, whereas may have investigated the short-term use of corticosteroids for patients with critical COVID-19-induced pneumonia [12] to delay a cytokine cascade and intercept the disease progression to a more serious form.

Medication with chloroquine and hydroxychloroquine offers mixed results. It can be detrimental to health because of cardiac toxicity. The World Health Organization (WHO) was the first to propose experiments using these two drugs considering their effects. There would seem, though, to be a need for basic clarification. Indeed, the paper was subsequently disowned, and the WHO relaunched the research on chloroquine and hydroxychloroquine, as stepped-up different issues concerning data and study carried out we identified and the reports were verified. Tocilizumab, a human, anti-interleukin-6-receptor (IL-6R) monoclonal antibody that delays interleukin-6 (IL-6) waving, is now being investigated in many clinical trials due to the excessive and abnormal immune response observed in COVID-19 to associated with ARDS and, in some instances, to fibrosis and extensive lung damage [13].

Preventive treatment and approach, self-defense, early diagnosis, and exclusion are the primary tools available to stop the prevalence and severity of the disease and inflammation it produces [14]. Several vaccines and pharmaceuticals are already being administered for treating COVID-19 after being tested for their efficacy, protection, and dosage verification. However, the pandemic goes on, the 4th wave is increasing, and it seems that this pandemic will never end.

Consequently, further options against COVID-19 can be structured by looking for the use/reuse of numerous available natural active compounds. A proven increased capacity with clear antimicrobial, antibiotic, anti-inflammatory results that boost immunity has already been demonstrated over time by innumerable pharmaceuticals and herbs, including essential elements, vitamins and compounds from other classes (such as zinc, vitamins C and D, curcumin, cinnamaldehyde, probiotics, selenium, lactoferrin, quercetin, etc.) [14,15]. Integration of any of these phytoconstituents (like vitamins) as a nutritional supplement) in the correct order may significantly boost the immune system, avoid the spread of the virus, prevent progress to the critical stage, and further inhibit the immune system hyperactivation providing prophylactic and therapeutic support against COVID-19 [16].

In the study of the related therapeutic outlook in many diseases, the pharmacological features of certain natural products have acquired growing recognition [17]. In this way, the food industry is attempting to manufacture substances that concentrate on bioactive substances found in food or in natural ingredients that are often regarded as nutraceuticals, which, besides their nutritional qualities, can provide results based on health benefits, in particular in the management of chronic diseases [18]. Compared to the standard drug treatments, these natural provenance therapeutics have minimal side effects, although no clinical trials or studies on the safety of non-pharmacological agents are available for the SARS-CoV-2 infection [19].

Supplements contain natural compounds such as vitamins, minerals, and proteins, etc, that are beneficial for human health and play an essential role in boosting the immune system, which can fight the COVID-19 virus and infection [20]. For this reason, we are trying to move towards these supplements and nutraceuticals that can eliminate the risk of infection by boosting the immune system and have no side effects. These are the best choices to fight against COVID-19 till any effective pharmaceuticals/drugs and vaccines can be manufactured and administered [21].

When the COVID-19 epidemic struck the world, one of the four aspects of food systems (food security, sustainable food development) directly influenced by the outbreak was the need for immune-supporting biologically active compounds [22]. Though panic buying and shortages [23] followed, customers generally loaded up on vitamin C and other herbal items. As a result, researchers and the medical profession have increased their efforts to examine drug ingredients that could support the immune system, protect against the acute respiratory virus, and prevents the growth of SARS-CoV-2, the latest coronavirus that induces infection with COVID-19, resulting in many comprehensive studies highlighting the potential of natural compounds to treat SARS-CoV-2 [24].

The downside to acquiring new insights from this expeditious strategy is that the resulting increase in recently reported research has yet to be exposed to any cost-benefit or effect analysis. Incremental developments promoting agri-food and emerging products are likely to be motivated by the immediate need for new health approaches for tackling COVID-19 and contributing to a rise in interest in accelerating innovations in the area of environmental and climate change [25,26]. Therefore, this current, timely analysis offers a thorough examination of the role of food and plant components and phytochemicals in early and fast-release experiments against the COVID-19 epidemic.

After an in-depth literature research, our team of authors have examined the potential role of nutraceuticals, plant-based products, and dietary supplements in viral infection with SARS-CoV-2, trying to highlight as best as possible the main compounds which are relevant in their action for the disease. Overall, more research is needed before evidence-based recommendations can be made, but nutritional status plays a big role in patient outcomes, and these supplements could help with that.

Vitamin D insufficiency, for example, has been linked to a higher prevalence of infection and severity of COVID-19, implying that vitamin D supplementation could be beneficial as a preventive or therapeutic measure [27]. Vitamin D supplementation is being considered by a rising number of scientific organizations for persons at high risk of COVID-19 [27]. We have also analyzed the extent to which vitamin D and other nutraceuticals and supplements offer a potential solution to the COVID-19 dilemma because research in these nutraceuticals and supplements is preliminary [27]. Herbal treatments are gaining popularity around the world as a way to treat several conditions. Before the discovery of antibiotics, herbal extracts were used to treat diseases, and purified natural products and herbal extracts now provide a rich pool of components for developing innovative antiviral medications [27].

Some studies have also discussed the essential importance of dietary additives and nutraceuticals after the recognition of the effects of long COVID-19 infections on survivors. For example, even in those that survive the initial infection, peripheral inflammation induced by COVID-19 can have long-term effects, contributing to chronic medical, psychiatric illnesses, and neurological disorders, possibly caused by neuroinflammatory pathways that can be exacerbated by an unbalanced lifestyle [27].

There is, therefore, an urgent need for broader access to healthier foods, and it is crucial to make people aware that good nutrition can reduce vulnerability to COVID-19 and its long-term consequences [28,29,30]. Nutrients are essential for the benefits nutritional opportunities and modes of action of food additives and bioactive components confer against the SARS-CoV-2 virus [31]. These bioactive components can be found in fruits and vegetables and their consumption in the daily diet is strongly advised [32]. Vitamin C can also, in such circumstances, reduce inflammation of the lower airways and avoid respiratory infections [33,34].

This review article highlights the most recent and relevant published results on the therapeutic potential of some promising natural products/compounds in terms of their activity and action against pathological agents such as viruses in general, and SARS-CoV-2 in particular.

## 2. Methodology

In order to succeed in this objective, the authors researched in depth the literature published in the field. In this regard, not only were those works (reviews and articles) that deal with the ideas of the present manuscript considered, but also those that underline the most important aspects of the topic were also selected. For complete and accurate information, the most well-known and common biological/medical databases (such as Cochrane Library, PubMed, Web of Science, etc.) were accessed and searched. The period in which the chosen articles were published and included as references citations was unlimited (until the date of submission of our paper), considering all the interesting ones. Both the terms Medical Subject Heading (MeSH) and the keywords already chosen and described (COVID-19; SARS-CoV-2; probiotics; herbs; nutraceuticals; supplements; therapy), but also combinations of them helped to find optimal articles and information. Initially, all eligible articles with the potential to be used in this research were chosen according to their keywords, title and/or abstract; subsequently, an analysis of the content was made by applying various filtering techniques as follows: publication language—English (this being known by all our team authors), the novelty of the information provided, etc.

**Figure 1:**
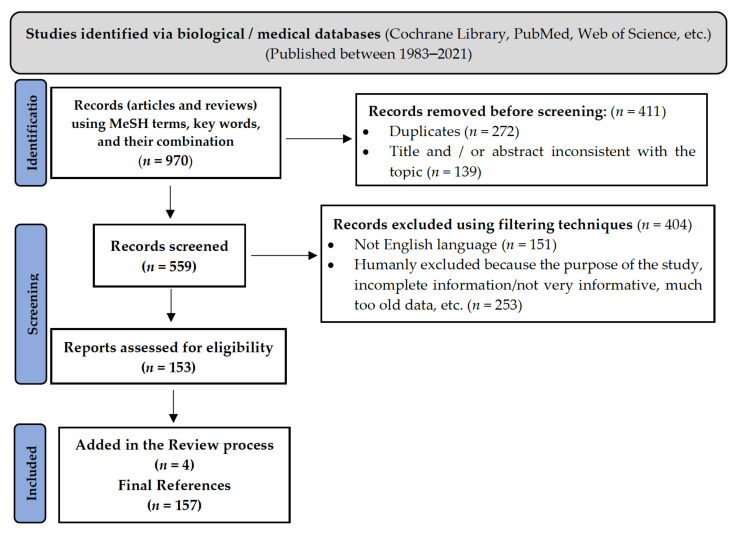
PRISMA flow-chart summarizing the criteria of selection of the literature. MeSH = Medical Subject Heading.

The criteria above are mentioned accordingly in a PRISMA flow chart (Figure 1), which summarizes the entire process of selecting the literature necessary to prepare this paper [35,36].

## 3. Characteristics of SARS-CoV-2

**Figure 2:**
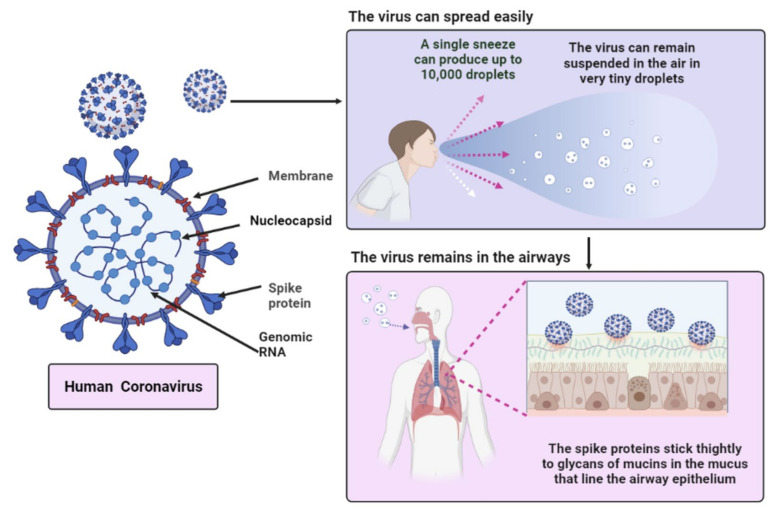
Structure and spread mechanism of the human coronavirus (Created with BioRender.com).

Coronavirus is a kind of virus with a circular or oval envelope that is sometimes pleomorphic. This virus has a diameter of 50–200 nm, and the genome of the coronavirus is 30,000 nucleotides long [37,38]. SARS-CoV-2 has notable, club-shaped spike-like projections on its surface, giving it the appearance of a solar corona. This novel coronavirus codes for four main structural proteins. Spike (S), membrane (M), envelope (E), and nucleocapsid (N) are the proteins involved (Figure 2), and the capsid is a protein shell that contains nuclear capsid, or N-protein, which is attached to the virus’s single positive strand RNA and allows it to hijack human cells and transform them into viral factories [38].

The N protein covers the viral RNA genome and is necessary for replication and transcription. In MHV and IBV virions, the N-terminal of the N protein binds to genomic and sub-genomic RNAs and processes viral replication and transcription [39]. The M-protein is most abundant in the viral surface, and it is believed to be the central organizer for the coronavirus assembly. Coronavirus is a contagious one that may be transferred by inhalation or contact. The most significant cause of infection is virus droplets inhaled because of coughing and sneezing as a single sneeze can produce up to 10,000 droplets as well as contacting a contaminated surface which remains in the air in very tiny droplet form as shown in Figure 2.

Also, the spike protein sticks tightly to glycans of mucins in the mucus that line the airway epithelium as S protein is integrated over the surface of the virus, it mediates attachment of the virus to the host cell surface receptors and fusion between the viral and host cell membranes to facilitate viral entry into the host cell. The E-protein is a tiny membrane protein with 76–109 amino acids and an essential constituent of the viral particle. It is involved in viral assembly, host cell membrane permeability, and viral-host cell contact [39].

## 4. Immune Response from Various Perspectives and Targeting SARS-CoV Viral Entry Mechanism

Human coronaviruses include SARS-CoV and the Middle East Respiratory Disease coronavirus (MERS-CoV) [39]. Two very different SARS-CoV and MERS-CoV are members of the genus that preceded the SARS-CoV-2. 229E and NL63, all from the family, and OC43 and HKU1 from the genus are the other forms of human coronavirus [40]. The RNA of this novel SARS-CoV-2 is 30 km long, the same duration as that of the SARS-CoV and MERS-CoV viruses that created problems many years earlier [41].

This novel virus shares 96% and 79.5% genome sequence similarities with a recognized bat coronavirus and SARS-CoV, respectively [42]. According to another report, this virus shares a genome-sequence resemblance of about 45% to 90% with SARS-CoV, but only about 20% to 60% with MERS-CoV compared to a recognized bat coronavirus and SARS-CoV. This SARS-CoV-2 has shorter gene sequences. However, it has a more extended sequence than MERS-CoV [43].

**Figure 3:**
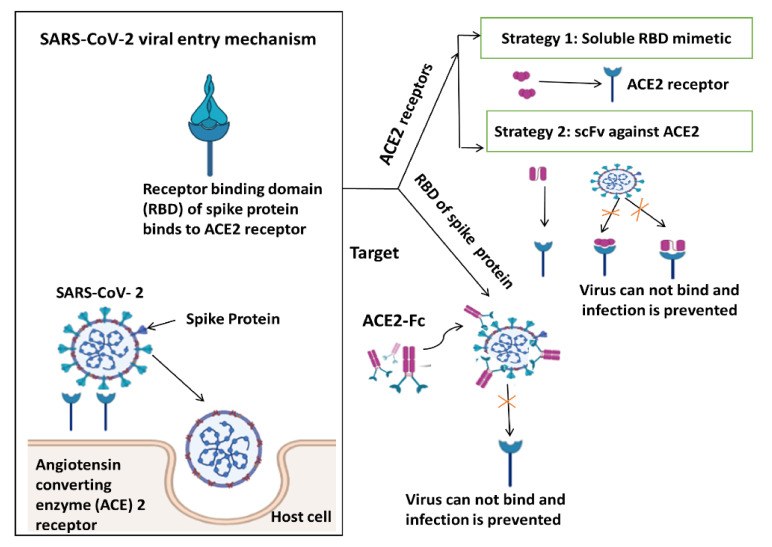
Targeting SARS-CoV viral entry mechanism. RBD, Receptor-binding domain; ACE2, Angiotensin-converting enzyme 2. (Created with BioRender.com).

For targeting SARS-CoV viral entry mechanism, there are two strategies to target the ACE2 receptor illustrated in Figure 3, the S protein of SARS-CoV-2, as well as SARS-CoV, binds to the human zinc peptidase ACE2, which is expressed on a variety of cells, including lung, heart, kidney, and intestinal cells, and thereby initiates viral entry into target cells [44]. S protein comprises two subunits, the N-terminal S1 subunit, which contains the receptor-binding domain (RBD), and the C-terminal S2 subunit, which is attached to the viral membrane and necessary for spike trimerization and virus-host membrane fusion [45]. During viral assembly, the host enzyme furin cleaves the S protein between S1 and S2. The membrane-bound host protease TMPRSS2 is in charge of the proteolytic activation of the S2’ site, which is required for conformational alterations and viral entry [46].

Another strategy is that antibodies against the spike protein of coronaviruses are being investigated as possible therapeutic candidates. Antibodies against the S1 subunit, notably against RBD, can potently neutralize SARS-CoV and MERS (Figure 2). SARS-CoV monoclonal human antibodies have been shown to cross-react with SARS-CoV-2. Some of them can also neutralize SARS-CoV-2 as one of the strategies. Several single-chain fragment variable-fragment crystallizable (scFv-Fc) antibodies effectively suppress SARS-CoV-2 infection by inhibiting spike protein binding to ACE2-expressing cells. The best IgG antibody might be a candidate for clinical development as passive immunotherapy for therapeutic and prophylactic purpose [47]. The targeting SARS-CoV viral entry mechanisms are shown in Figure 3.

## 5. Nutraceuticals or Supplements Targeting COVID-19

The term “nutraceuticals” is a mixture of two terms, “nutrient” and “pharmaceutical”; nutrient means a highly nutritious food component and food item and pharmaceutical means a medical drug. DeFelice, founder and chief executive of the Foundation for Medicine Intervention, a U.S. association founded in Cranford (NJ) proposed and used the term for the first time in 1989 [48].

Nutraceuticals reflect the general biological therapy and are used to treat extremely damaging malignancies with minor disordered symptoms [48]. Several healthcare authorities have issued compassionate use approvals the onset of the 2019 COVID-19 disease outbreak of corona infectious disease [49]. Apart from remdesivir, which displayed promising effects and received urgent approval by the Food and Drug Administration (FDA) for use in the treatment of COVID-19, no clear antimicrobial medication has yet been reported to be successful [50].

It has been proposed that an essential feature of COVID-19 is that an acute alveolar and interstitial inflammation spreads into the pulmonary vasculature [51]. The resulting vital local vascular disorder, comprising micro-thrombosis and hemorrhage and culminating in pulmonary intravascular coagulopathy (PIC) [52] is adversely influenced by the severity of the intra-pulmonary infection. The reduced endothelial activity, which is an established subclinical phase of cardiovascular alteration, could also support the progression of a mortal form of this disease, resulting in increased coronary, cardiac, and renal abnormalities [53].

After the respiration mechanism, the immune response is the second thing impaired by COVID-19. During the rapid development stage of COVID-19, development of increased levels of circulating interleukins, chemokines, and tumor necrosis constituent-alpha (TNF-alpha) was studied. Cytokine release syndrome (CRS), in which several of the highest priority benefits from IL-6. In a recent back into the past multicenter analysis that shows that circulation IL-6 levels were higher in departure COVID-19 cases assigned to the discharged substances, the function of IL-6 in COVID-19 patients has been established [54]. 

These findings indicated that the viruses might be a medicinal indicator of lethal impact in these patients that would get the cytokine storm syndrome. Other traditional characteristics of essential CRS are the stimulation of endothelial cells and an evolving vascular disease. The average endothelial awakening markers are commonly elevated in the serum of CRS patients. This indicates that endothelium contributes to the anatomy of the CRS pathway, both by producing the inflammatory response and by spreading it to clotting, and eventually to thromboembolic (coagulum) disorder, and both in arterial and venous circulation [55].

Nutraceuticals, identified as an alternative to pharmaceuticals that may include nutritional supplements, derived nutrients, dietary and herbal products, display physiological advantages that may play a significant role in reversing inflammatory cataract sensitivity and hypercoagulation through prescribing anti-inflammatory and antioxidant medications, based on these reviews and findings [56]. Vitamins C and E, carotenoids, certain elements (like Zn, Mn, Cu, and Se), and polyphenols like flavonoids, phenolic acids, stilbenes, and lignans, provide therapeutic or health advantages by working together to maintain an effective redox (oxidation-reduction) homeostasis [57]. Mainly, a polyphenol-rich diet is ideal for minimizing and avoiding cardiovascular disease. In addition, the shielding impact of polyphenols includes a reduction in oxidative stress or pressure arising from the inhibition of nicotinamide adenine dinucleotide phosphate (NADPH) oxidase or anti-platelet and anticoagulant work, as defined by the decrease in platelet aggregation, the suppression of thrombin activity, and the existence of factor Ax [58].

In addition, flavonoids could strengthen internal platelet-derived nitric oxide, decrease the development of superoxide, increase nitric oxide (NO)’s endothelial structure, convince NO-depending on relaxing or stimulation in separated arteries, and initiate NO signaling roads in endothelial cells, thus improving endothelial activity. In the end, several polyphenols allow every attempt to regulate the cytokine formation mechanism in anti-inflammatory roles by facilitating the appearance of pro-inflammatory genes and antiviral activity that has already been recorded against many viruses [59].

In brief, till now, the accumulated literature has demonstrated that in the situation of vascular diseases resisting oxidative disruption and proliferation, nutraceuticals exert beneficial results; it is fair to hypothesize their practical application in the circumstances of COVID-19 [60]. Dietary supplements are products extracted from animals, plants may be synthesized by enzymes and are taken as pills, capsules, in powdered form and maybe in liquid form and also extracted from food sources or artificially produced to increase their quantity available for consumption [61]. They include one or more dietary elements such as vitamins, herbs, amino acids, enzymes, and minerals, etc. Out of all supplements, the most consumed multivitamin supplements are omega-3 or fish oil, vitamins C and D, and calcium [62].

Phase I is a critical time for prevention because, in this phase, the person is a carrier that can spread the infection unintentionally. The patients from this phase must manage themselves with prepared specific extensible immune responses and should use antiviral drugs that can stop the entry of virus and replication and the disease progression to phase II. During phase II, along with the victim’s health condition, the procedure must focus on the adaptive strategies that include the use of supplements to overpower the oxidative trauma, cytokine storm, and acute inflammation so that damage caused to affected tissues is averted [62].

Several types of research have proved that taking any food supplements and nutritional supplements that are obtained from different species such as herbs, roots, fruits, and vegetables, etc. have an effective result on health by boosting the immune system, particularly in those who have insufficient diet sources, and they have any anti-inflammatory, viricidal and radical scavenging features and these compounds should be recycled to reduce the adverse results that are reported after contamination with SARS-CoV-2. Subsequently, the use of natural products can provide complementary therapeutic and prophylactic help to prevent COVID-19 side effects [63].

### 5.1. Vitamins

#### 5.1.1. Vitamin C

Vitamin C is water-soluble and essential for both the natural and extended immune system; there are many bits of evidence in in-vitro studies that ascorbic acid (as it is also known) has antiviral effects [22]. It has been reported that a high dose of vitamin C can destroy viruses because it has a virucidal impact that it can inactivate the multiplication of viruses in vitro, but this virucidal effect of vitamin C has not been confirmed in vivo [64]. The beneficial antiviral impact of this vitamin is demonstrated in many clinical studies [65]. Vitamin C supplements in humans improve the immune system, protect from reactive oxygen species (ROS), and reduce the risk, duration, and intensity of many infectious diseases [66]. Additionally, the deficiency of vitamin C is associated with pneumonia, as it confirmed in old research and studies.

Furthermore, published data and demonstrations have shown and confirmed that vitamin C supplements positively affect the duration and symptoms of different respiratory tract infections [66]. The latest findings (Table 1) indicate that vitamin C has an enormous impact on essential viral disease, lacking any particular treatment for COVID-19. In addition, vitamin C can increase tolerance to the coronavirus and, under specific circumstances, can influence the susceptibility to lower respiratory tract infections [66].

#### 5.1.2. Vitamin D

Vitamin D’s role in the metabolism of the bone is well recognized [67]. Many experiments have shown that there are many biochemical pathways in which vitamin D can enhance the autoimmune response and reactions, regulate chronic inflammation, and reduce the danger to the human psyche of disease in a helpful manner [67,68]. Amon the many nutrients naturally present in food, the quantity of vitamin D available for intake is insufficient and for this reason, the food must be fortified, and orally taken supplements are often necessary to fulfill the body’s vitamin D requirement [69].

Several studies have shown that high vitamin D levels correlate with improved prognosis and are successful in treating infectious diseases. It has been verified in recent reports that vitamin D supplements reduce the likelihood of acute respiratory infections, as confirmed in many clinical studies (Table 1) [70]. In large-scale randomized studies, the presumption that vitamin D supplementation will reduce the likelihood of COVID-19 incidence, or the ratio of death should be evaluated or tested [71]. Currently, no details on dose, regular or tablet control methods (bolus), and security in the treatment of COVID-19 are available. However, scientists must focus on the detection and therapy of symptomless subjects or difficulties in victims afflicted by COVID-19 [72].

### 5.2. Minerals—Zinc

Some micronutrients such as folic acid, zinc, vitamins, and iron are essential, boosting our immune system [73]. Increased intracellular zinc concentrations efficiently impair replication in several RNA viruses [74]. Zinc has been shown to enhance cytotoxicity and induce apoptosis when used in vitro with a zinc ionophore (e.g., chloroquine). Chloroquine has also been shown to improve intracellular zinc uptake in vitro [75]. The relationship between zinc and COVID-19, including how zinc deficiency affects the severity of COVID-19 and whether zinc supplements can improve clinical outcomes, is currently under investigation [76]. Zinc levels are challenging to measure accurately, as zinc is distributed as a component of various proteins and nucleic acids [76]. Several clinical trials are currently investigating the use of zinc supplementation alone or in combination with hydroxychloroquine to prevent and treat COVID-19. The recommended dietary allowance for elemental zinc is 11 mg daily for men and 8 mg for nonpregnant women (Table 1). The doses used in registered clinical trials for patients with COVID-19 vary between studies, with a maximum dose of zinc sulfate 220 mg (50 mg of elemental zinc) twice daily [77]. However, there are currently insufficient data to recommend either for or against the use of zinc to treat COVID-19.

### 5.3. Probiotics

Probiotics are nonpathogenic living microorganisms providing various health benefits to their human host [78]. These include the *Lactobacillus* and *Bifidobacterium* genera of bacteria, typically present in fermented food. Traditionally prepared cuisine often includes such fermented foods since these bacteria restore the balance of microbes in the gut [75]. Over the last two decades, many studies and clinical trials have suggested that probiotics may help modulate the immune response and treat various diseases, especially viral infections [79]. Many findings indicate that such probiotics maintain a healthy host immune system that helps the body rebound after a respiratory viral infection in animal models; not only did these interventions enhance the health of the animals, but they lowered the viral load in their lungs and boosted survival rates [80]. These arrived at a rough list of probiotic strains that may help prevent infection and enhance immune function to reduce the impact of viral infections, especially COVID-19 [81]. The SARS-CoV-2 virus may be transmitted even in asymptomatic individuals or during the pre-symptomatic phases of the disease. The latter may last for up to 2 weeks, exposing others to heavy viral loads and a high risk of infection [81] as demonstrated further in Table 1. Probiotics may trap the virus in respiratory diseases and inhibit the binding of the virus to the host cell receptors [9].

### 5.4. Natural Polyphenols

#### 5.4.1. Resveratrol

Resveratrol (RSV), a polyphenolic compound, chemically known as *trans*-3,5,4′-trihydroxystilbene is found in red grapes, berries, peanuts, and bamboo [82]. Several studies have shown that it has antitumor activity, antioxidant activity and potent antiviral effects, anti-ageing properties, and cardioprotective effects in-vivo and in-vitro [83]. The therapeutical efficacy of polyphenols has been carefully vetted in the control and diagnosis of retroviruses and, in particular, in respiratory problem-causing microbes [84]. Wahedi et al. [85] have shown polyphenols to form an extremely reliable compound with the human ACE-2 transmitter using MD simulation stimulus, which COVID-19 needs to reach target cells using S-protein. Various reports also show an improved intake of supplementary RSV, related to higher plasma level, when eaten with food, whereas others document a slower but not radically changed cumulative absorption.

Published data reported that RSV’s antiviral effectiveness has been demonstrated against a variety of viruses, like coronavirus, and it has been shown to block the critical pathways involved in SARS-CoV-2 pathogenesis, including control of both RAS and ACE2, body immune activation, and downregulation of pro-inflammatory cytokines release. It was also discovered to activate SIRT1 and p53 signaling pathways and increase cytotoxic T lymphocytes (CTLs) and natural killer (NK) immune cells. RSV has also been a fetal hemoglobin stimulator and a potent antioxidant capable of blocking ROS [86,87].

RSV administration showed antiviral effects against various viral pathogens in both in vivo and in vitro trials [84]. It also blocked MERS-CoV replication in vitro by inhibiting RNA synthesis and had other pleiotropic consequences, and it inhibited viral replication and reduced the death risk in piglets infected with the duck enteritis virus [84]. Although RSV has a low bioavailability, nanoparticle formulations can improve its stability and absorption [88,89]. There is currently no evidence that RSV has been used to treat SARS-CoV-2; nevertheless, available research suggests that it may be a beneficial adjunctive antiviral agent to consider.

#### 5.4.2. Quercetin

Quercetin is a flavonoid that is the most abundant in vegetables and fruits [90]. The compound has antioxidant, anti-inflammatory, immune system control, cardioprotective, and neuroprotective properties. Quercetin’s antioxidant potential and free radical scavenging ability are primarily responsible for its neuroprotective impact [91]. Supplementing with quercetin has been shown to improve mitochondrial biogenesis, energy output, and electron transport chain performance and modify ROS production and mitochondrial defects. It can cross the blood-brain barrier [92].

A randomized, open-labelled, and controlled study aimed to investigate the adjuvant benefits of quercetin phytosomes in community-based subjects with confirmed SARS-CoV-2 infection (by RT-PCR). The study has two arms. In one arm, the subjects will receive standard COVID-19 care per the hospital/physician guidelines, whereas in the other, the subjects will receive routine COVID-19 care + quercetin phytosomes. The treatment will continue for 30 days. It is proposed that quercetin phytosomes will boost the natural immunity of the subjects and help prevent the COVID-19 disease progression, i.e., avoiding the need for hospitalization [93]. Quercetin may thus be a novel therapeutic agent for COVID-19-induced AKI. Inhibition of inflammatory, cell apoptosis-related signaling pathways may be the critical mechanism by which quercetin protects the kidney from SARS-CoV-2 injury [94,95] and is mentioned in Table 1.

## 6. Herbs Considered Usable for the Treatment of COVID-19

### 6.1. Curcuma longa

*Curcuma longa* (*C. longa*) is a therapeutic rhizomatous plant of the *Zingiberaceae* family known as turmeric. It has piqued the interest of medical and scientific societies due to its enormous health benefits [96]. In Asian countries, *C. longa* has long been used as a prescription medicine or supplement to treat diabetes, coronary disease, obesity, neurodegenerative disease, inflammatory bowel disease, allergy or asthma, and psoriasis [97]. It also serves as an antioxidant, anti-inflammatory, and anticancer agent. *C. longa* contains carbohydrates (96.4%), proteins (6.3%), fat (5.1 %), minerals (3.5%), and moisture (3.5%) (13.1%). Its extracts contain curcuminoids, including curcumin (77%), desmethoxycurcumin (DMC; 17%), and bisdemethoxycurcumin (BDMC; 3%). Curcuminoids, particularly curcumin, are used as medicines and supplements, being also an effective therapy for hypertension as demonstrated in several trials [21,98]. On the other hand, a comprehensive study found that using curcumin as more of an antihypertensive requires a longer time to impact blood pressure reduction, about 12 weeks [99].

A renin-angiotensin-aldosterone system (RAAS) inhibitor (such as ACE inhibitor) increased ACE2 expression in a preclinical study. Another study, on the other hand, found that administering a RAAS inhibitor had little effect on the ACE2 receptor [100]. Based on these findings, it’s possible that using curcumin to avoid COVID-19 infection might result in a highly susceptible person being infected with SARS-CoV-2 and worsening their condition. Since the novel virus has a distinct receptor, ACE2, which is also active in the hypertension pathway, this phase happens. Curcumin, as a RAAS inhibitor, has been shown to improve the expression and synthesis of ACE2. As a result, using curcumin to prevent infection can trigger susceptible people to contract COVID-19. Curcumin as a COVID-19 therapy may be reconsidered since it induces a rise in ACE2, accelerating the disease’s development [101].

Curcumin has been used as an immunomodulator for both immunostimulation and immunosuppression [102]. It may raise or decrease pro-inflammatory cytokines. CRS, or cytokine storm, is a hyperinflammatory and hypercytokinaemia seen in some COVID-19 patients [103], but curcumin can also increase the development of pro-inflammatory cytokines, worsening the status of COVID-19 patients who are experiencing a cytokine storm. Further research is needed to understand further the function of curcumin in the prevention and treatment of COVID-19.

### 6.2. Cinchona officinalis

Cinchona plant from Andean Mountain forests provides substantial benefits since a particular portion of the trees produce bioactive compounds that may help cure fever, and Jesuit missionaries were the first to notice this beneficial influence, which spread across the world [104]. Quinine is a plant-derived medicine currently under investigation for COVID-19 treatment that was discovered as the principal bioactive compound in *Cinchona officinalis*, a south American antimalarial medicinal plant [105]. A preliminary clinical study in France recently showed promising activity of chloroquine and hydroxychloroquine in reducing the SARS-CoV-2 viral load in COVID-19 patients. Interestingly chloroquine and hydroxychloroquine are synthetic analogues of quinine and have been approved for treating malaria, human immunodeficiency virus (HIV), systemic lupus erythematosus, and rheumatoid arthritis [106].

According to a clinical study, when administered with azithromycin, hydroxychloroquine reduces the SARS-CoV-2 viral load in COVID-19 patients, and this evidence suggests that quinine, as a chloroquine analogue, could also have beneficial effects in eliminating COVID-19 [107]. The second plant-derived example is the lung cleansing and detoxifying decoction (LCDD), widely used in treating COVID-19 patients in China that was developed based on four formulae described in the classic text Treatise on Cold Pathogenic and Miscellaneous Diseases by Zhang Zhongjing [108].

### 6.3. Allium sativum

For decades, the beneficial medicinal effects of *Allium sativum* (garlic) have been known. It comprises several compounds that can affect immunity [109]. Recent studies have studied its potential active compound allicin as a possible candidate for strengthening the immune system, and the immune regulatory roles of garlic extracts and extracted compounds were thoroughly investigated [110]. Immune dysfunction is well-known to play a role in the development and progression of various diseases, and this functional diet additive can help prevent and treat diseases including obesity, metabolic syndrome, cardiovascular disorders, gastric ulcers, and even cancer in treating cancers affected by compounds like aflatoxin B1. Aged garlic extract (AGE) may be used as an herbal remedy with fewer side effects than chemotherapy [111]. It also seems to mitigate the impact of COVID-19 infection, and this functional food, when used as part of a preventive strategy can strengthen immune system components in the battle against this pathogen [112]. Pre-existing cancer and/or hypertension are considered two of the main factors leading to increasing morbidity and mortality in COVID-19 infection; garlic is considered as having essential role in these conditions as well [108].

### 6.4. Sambucus nigra

*Sambucus nigra* belongs to the *Adoxaceae* family, commonly known as elderberry and is a kind of berry native to the Mediterranean region, Asia, and North Africa [113]. In herbal formulations, both the rose and fruit of mature trees have traditionally been used to treat influenza [114]. The bioactive compounds found in elderberry are primarily polyphenols and anthocyanins. The primary polyphenols in elderberry fruit are chlorogenic acid, neochlorogenic acid, cryptochlorogenic acid, quercetin, quercetin-3-rutinoside (rutin), quercetin-3-glucoside (isoquercitrin), kaempferol-3-rutinoside, kaempferol-3-glucoside (astragalin), isorhamnetin-3-rutinoside and isorhamnetin-3-glucoside. The primary flavanol in this plant is rutin, while the other flavanols, like iso-quercitrin and astragalin, occur in elderberries in smaller amounts [115,116]. Its extracts were used in the early stages of the epidemic, although its supplements have not been tested for coronavirus treatment, randomized, double-blind, placebo-controlled trials and meta-analyses have shown that they effectively treat colds and influenza [117]. There have been extensive reports on the beneficial effects of elderberry products on various viral infections. Few theories have been established on the potential immunological mechanisms for elderberry supplements beneficial effects as they may help reduce upper respiratory symptoms caused by viral infections and help alleviate flu symptoms [118].

### 6.5. Piper nigrum L.

*Piper nigrum L*., the king of spices, is also known as black pepper, Kaali Mirchowing, due to the widespread usage of its dried, freshly selected fruit in nearly all cuisines worldwide [119]. Apart from that, this plant produces over 600 different bioactive compounds, including lignans, terpenes, and neolignanes, all of which encompass different bioactive compounds and therapeutic applications. The anti-parasitic biological function of black pepper was studied in depth [120]. The main alkaloid constituents, piperine and piperamides, are phytochemicals that can aid in the battle against COVID-19, where it appears to reduce respiratory system infection and have antiviral and antibacterial properties [121].

### 6.6. Withania somnifera

*Withania somnifera* (WS), also known as Ashwagandha, is one of the most extensively researched medicinal ayurvedic plant extracts, and it has been used for decades in the Ayurvedic tradition. There is currently no effective pharmacological prophylaxis against COVID-19 [122]. For millennia, Ashwagandha has become one of the most widely used Rasayana herbs in the ayurvedic tradition. WS has regenerative, adaptogenic, immunomodulant, therapeutic adjunctive, and vaccine anticoagulant properties [123].

Another Rasayana Ayurveda contains several plant ingredients along with WS that may boost the immune system and can be helpful in the treatment and prevention of COVID-19. Rasayana therapy involves the application of medicines, diet, and behavior. Many clinical and laboratory studies have shown that Rasayana is effective in immune modulation, rejuvenation, regeneration, adaptogenic, and immune homeostatic function reconstruction [124]. When developing diagnosis and clinical goals, it is critical to understand the causes of COVID-19 and its common lung affliction before the internal organ collapse and neurological abnormalities that often precede death occur [125]. WS has been shown to have several beneficial pulmonary consequences, including lowering pulmonary hypertension through decreasing inflammation, oxidative stress, epithelial dysfunction, respiratory cell apoptosis, and pulmonary respiratory system tolerance [126]. WS has been shown to trigger the innate immune system and selectively regulate the immune response of Th1 cells. The antiviral properties of the phytoconstituents of WS have previously been demonstrated in experiments. The transforming enzyme of angiotensin has also been shown to be suppressed by an aqueous WS extract, which is now being seen as a potential therapeutic target for COVID 19 therapy [127,128].

### 6.7. Ocimum sanctum

*Ocimum sanctum*, also popular as Tulsi or holy basil, is an Indian wild plant known for its medicinal properties in the ayurvedic and siddha therapeutic frameworks [87]. Several in-vitro and in-vivo experiments on humans and other species have shown their therapeutic properties as antibacterial, antidiabetic, anti-carcinogenic, antiviral, bashing, cardio-protective, immune system enhancer, and so on [87]. Tulsi is recognized in Ayurveda as the ‘Elixir of Life’ because of its powerful curative properties and ability to cure various ailments, including lung disease, vertigo, rheumatoid arthritis, fibromyalgia, skin infections, urinary and infectious diseases, gastrointestinal and pulmonary diseases, and more [129]. In terms of *Ocimum sanctum* function in COVID-19 management, Tulsi is often used to relieve pain, diarrhea, coughing, and congestion, all of which are common COVID-19 symptoms [41,130,131].

In an in-silico study of the phytochemicals present in Tulsi, primarily flavonoids and polyphenolic acids, it was revealed that they could be M^pro^ inhibitors. Studies have shown these phytochemicals to be efficient antiviral agents against various viruses [129]. The lead molecules are chlorogenic acid and luteolin-7-O-glucuronide. Coffee beans have the highest concentration of chlorogenic acid. SARA CoV 3CL (homologous to M^pro^ SARS CoV2) is inhibited by flavonoids [129]. 

### 6.8. Astragalus membranaceus

*Astragalus membranaceous* (AM) roots are used in various Indian and Chinese ayurvedic medicines that help improve the immune system and combat diseases. This herb is also a natural supplement to help with stress relief, tumor cell death, and chemotherapy side effects. Polysaccharides in *Astragalus* that produce mannose, D-glucose, xylose, and L-arabinose on hydrolysis have been found to have anti-inflammatory, antimicrobial, and antiviral properties [132]. *Astragalu*s polysaccharides as one of the main components of AM has been proven to be an immunomodulator and prevent viral infection in chickens infected with the H9N2 avian influenza virus in an in-vitro/in-vivo analysis published in 2013 [129]. IL-12, LITAF, IL-10, IL-6, IL-4, and antibody titers to H9N2 AIV were shown to be higher in the first week after therapy during in-vivo lymphocyte and in-vivo antibody titer study [133].

### 6.9. Nigella sativa

*Nigella sativa* (NS) is known by several names, including black cumin, black seed, and kalonji, and has been used to treat a wide range of ailments, including jaundice, conjunctivitis, rheumatism, diabetes, anorexia, stomach complications, intrinsic hemorrhage, asthma, cough, pneumonia, fever, bronchitis, pneumonia, and more [133]. Thymoquinone, the most powerful phytochemical in black cumin, is responsible for the bulk of the spice’s therapeutic properties [134]. NS oil and seeds have been shown to be virucidal against a number of lethal viruses, including HCV and HIV [135]. NS seed oil suppressed the viral burden to undetectable levels within ten days of intraperitoneal (i.p.) injection of black seed oil in a murine model [136].

In another study utilizing molecular docking, the authors uncovered new potential COVID-19 inhibitors using compounds from *Nigella sativa* L., a well-known therapeutic plant in North African communities and Islamic and Christian traditions. The discovery of the Mpro protease structure in COVID-19 opens many possibilities for finding new therapeutic options. Docking of chemicals from *Nigella Sativa* and medications under clinical test was performed using Molecular Operating Environment software, with a focus on the primary proteases in CoVs (3CLpro/Mpro) (PDB ID 6LU7 and 2GTB) (MOE). Nigelledine docked into the 6LU7 active site produces an energy complex of about −6.29734373 Kcal/mol, which is like chloroquine’s (−6.2930522 Kcal/mol) and better than hydroxychloroquine’s (−5.57386112 Kcal/mol) and favipiravir’s (−4.23310471 kcal/mol). Docking into the 2GTB active site revealed that α-hederin has an energy score of around 6.50204802 kcal/mol, which is higher than those of chloroquine (−6.20844936 kcal/mol), hydroxychloroquine (−5.51465893 kcal/mol), and favipiravir (−4.12183571 kcal/mol). The strongest candidates for COVID-19 treatment thus appear to be nigellidine and α-hederin. Further studies are needed to confirm the medical value of *Nigella sativa* and to advocate its usage as a preventative measure against coronavirus infection [136].

### 6.10. Tinospora cordifolia

*Tinospora cordifolia*, also known as giloy or guduchi, is one of the most adaptable restorative plants. It is a common herb used in Ayurvedic medicine. It is regarded as one of the strongest Rasayanas and is notable for its remarkable versatility [137]. Many biologically essential phytochemicals, such as lactones, alkaloids, glycosides, hormones, sesquiterpenoids, diterpenoids, aliphatic compounds, phenolics, polysaccharides and flavonoids, display immunomodulatory activity in the human body It has anti-diabetic, antioxidant, anti-inflammatory, antiperiodic, antispasmodic, anti-arthritic, anti-allergic, antimicrobial, anti-osteoporotic, antitoxic, anti-stress, anticancer, anti-HIV, wound healing, cardiotonic, carminative, bitter tonic, blood purifier properties that help to improve digestion and boost the immune system, as well as broad-spectrum antimicrobial effectiveness against *Staphylococcus aureus*, *Klebsiella pneumonia*, *Escherichia coli*, *Shigella flexneri*, *Salmonella paratyphi*, *Salmonella typhimurium*, *Salmonella typhi*, *Enterobacter aerogene, Pseudomonas aeruginosa*, *Serratia marcesenses*, and *Proteus vulgaris* activity as demonstrated in one study [87,138,139,140]. Tinosporin, tetrahydropalmatine, choline, palmatine, and magnoflorine are among the alkaloid components that guard against aflatoxin-induced nephrotoxicity [141]. Only one compound, namely tinocordiside (CID_177384) showed a high binding affinity as compared to built-in ligand N3 for SARS-CoV-2 M^pro^ as per YASARA scoring. Tinocordiside had a binding energy of 8.10 kcal/mol and was found to be a new rearranged cadinane sesquiterpene glycoside [142].

### 6.11. Glycyrrhiza glabra

Glycyrrhizin, the most bioactive ingredient of *Glycyrrhiza glabra*, has been shown to interfere specifically with ACE2, implying that it may be used as a COVID-19 treatment. The antiviral potency of ribavirin, 6-cytidine, pyrazolfurin, serum cholesterol, and glycyrrhizin against dual coronavirus strains (FFM-1 and FFM-2) was investigated in the hunt for antiviral drugs to treat SARS [143]. Among the compounds tested, glycyrrhizin was perhaps the most effective at inhibiting FFM-1 and FFM-2 virus replication [144]. Glycyrrhizin not only stopped FFM-1 and FFM-2 viruses from replicating but also disrupted their biosorption and osmotic replication cycles [145]. Moreover, the crude extract has anti-herpes simplex virus 1 (HSV-1) action, which may be due to its good anti-adhesion properties, which specifically inhibited HSV-1 virus attachment [143].

### 6.12. Zingiber officinalis

Zanjabeel or adrak is also known as *Zingiber officinale* Roscoe. Among others, it’s also an important medicinal plant that belongs to the family of *Zingiberaceae*. Its chemical constituents include 1–3% weight of volatile oils, which are responsible for its unique flavor and fragrance and zingerone and gingerols [146]. These chemicals have antimicrobial activities against various bacteria, viruses, and fungus. Especially the presence of one of the antioxidant compounds in the ginger root has potential anti-inflammatory and immune-boosting characteristics, which helps enhance the normal metabolic activities in the human body, fight against infections and toxins to shield against harmful effects of bacteria, viruses, and any other diseases. Thus, ginger has proved to be a powerful immune booster plant with particular antiviral properties [130]. In comparison to dried ginger, the fresh one is reported to inhibit human respiratory syncytial virus (HRSV)-induced plaque formation when taken in proper doses in both the adenocarcinomic human alveolar cancer cell line A549 and human laryngeal carcinoma (HEp-2) cell lines. Also, 300 µg/mL of fresh ginger is found to reduce the plaque counts to 19.7% (A549) and 27.0% HEp-2 compared to the control group, respectively [130]. It shows more promising results when given before viral inoculation [130].

The various discussed herbs/plants are mentioned in Table 2 along with their biological sources, diagram, family, active phytoconstituents with chemical structures along with their role or interventions in combating COVID-19 or for prevention from viral infection.

As scientific data has proved hundreds of times, it is obvious that medicinal plants are real “reservoirs” rich in bioactive substances. They often manifest numerous activities, sometimes pleiotropic, and can be further used with encouraging results as nutraceuticals (in prevention) and therapeutic strategies as well. Immunomodulators are a good example of plant-based nutraceuticals, being endowed with good potential as immune system stimulants, counteracting infectious and/or exogenous lesions, with immunosuppressive effect in controlling abnormal immune responses due to autoimmune diseases or/and as adjuvants contributing to modulation of nonimmune targets [154].

Additionally, natural products and herbal medicines have been historically used for acute respiratory infections, generally showing acceptable toxicity. The favorable stability for oral formulations and ease of scaling up manufacture make them ideal candidates for prophylaxis. Recently, many natural products have been reported to show inhibitory effects on human coronavirus. Natural products due to their unique carbon skeletons tend to inhibit viruses by acting on different targets [92,155]. A flavone compound (scutellarein) originated from *Scutellaria lateriflora* potentially inhibited the SARS-CoV helicase protein in-vitro through arresting the adenosine triphosphatase (ATP-ase) activity of nsP13 [156]. Another study has shown that two polyphenols derived from black tea, tannic acid and theaflavin, have shown potent activity against SARS-CoV by targeting chymotrypsin-like protease (3CLPro), an enzyme responsible for proteolysis and vital for coronavirus replication [157]. A group of compounds derived from rhizomes have showed anti-viral activity against SARS-CoV-2 by targeting the papain-like proteases, that cleave the viral polyproteins which are essential for its survival and replication [92].

Vaccines are agents that act in the human body and trigger the immune response to recognize and fight against disease-causing organisms. In some disorders, the vaccines alone are not strong enough to protect the body from infection; so, in these cases, multiple strategies have been used and must be tried to boost the power of vaccines. Studies show that natural products can act on several targets to prevent SARS-CoV-2 infection. Therefore, if these plan-based phytochemicals are administered in combination with vaccines, there is a high probability that they will strengthen and potentiate the ability of vaccines to be effective in the fight against pathogens.

## 7. Conclusions and Future Perspectives

To date, the number of allopathic medicinal products considered to be effective against COVID-19 is very limited. However, both dietary and plant-based medicine offer an alternative with recognized potential, very good, effective, and easily accessible to patients suffering from COVID-19 or in the recovery period after the disease.

Even though the supplements and nutraceuticals do not directly impact the treatment of viral infectious diseases, many studies show that taking these products and supplements can boost our immune system, which can improve the feedback of viruses and balance the inflammatory reactions. SARS-CoV-2 affects the gastrointestinal tract, which can cause inflammation of the mucosa and occasionally cause diarrhea. According to the updated evidence, the supplements taken orally play an essential role in the systematic and intestinal effects of COVID-19. In addition, the herbals play a crucial role in combating COVID-19 since they have antiviral effects, so they boost the immune response and prevent or reduce viral infections. They also have antioxidant and anti-inflammatory properties, which make the phytoconstituents present in herbs a promising approach to develop drugs and the beneficial effect of consuming it raw or in extract form to some extent.

Boosting the immune system is the only long-term way to live in the current environment. Antiviral and immunomodulatory activities can be found in various foods and herbs—gene expression, cell activation, and signaling molecule modification influence a well-balanced diet and food consumption. Furthermore, different food items influence the gut’s microbial makeup and, as a result, the immunological response in the body. This protection can be considerably enhanced by combining a diet with medicinal plants with immunomodulatory, antiviral, and anti-inflammatory properties. Vitamins C, D, E, zinc, and polyunsaturated fatty acids, among others, have an immunomodulatory effect too. Dietary supplements have been proven in recent research to have favorable benefits, such as lowering the viral load of SARS-CoV-2 and shortening the recovery time in COVID-19 patients.

Published clinical data are still partially inconclusive; also, inconsistencies can be observed in these reported data, because the existing clinical trials are few, and in some cases, they did not provide the desired effects and the expected results. It is suggested that the inconsistencies mentioned above could be correlated to several factors (i.e., heterogeneity of the studied population, dose of product used in therapy, plasma concentration values, route/mode of administration, duration of treatment, etc.).

With all the attention focused on these compounds in the context of the current pandemic evolution, it is obvious the need for additional experimental and clinical studies (in vitro and in vivo), performed as soon and quickly as possible, to validate the usability of natural products for drug treatment in COVID-19, in order to validate their effectiveness and to counteract the increase in the number of cases.

## Figures and Tables

**Table 1:** Supplements their role in COVID 19 treatment with daily requirement and clinical trial status.

Supplements	Daily Requirement	Intervention	Clinical Trial Phase	Reference
Vitamin C	250 and 1000 mg	Reduces the duration and severity of upper respiratory infections (viral infections). Scavenges ROS, prevents lipid peroxidation, and protein alkylation and thus protect cells from oxidative Decreases pro-inflammatory cytokines, TNF-α and IFN-γ and increases anti-inflammatory IL-10 production.	Phase II	[22,65,66,67,68,69]
Vitamin D	1000 to 4000 IU of supplemental vitamin D per day	Blocks NF-κB p65 activation via up-regulation of I-kappa-B-alpha (IKB-α). Decreases the expression of the pro-inflammatory type 1 cytokines: IL-12, IL-16, IL-8, TNF-α and IFN-γ Increases type 2 cytokines IL-4, IL-5, IL-1	Phase II	[70,71]
Zinc	11 mg for men and 8 mg for non-pregnant women, daily	Protects against oxidative stress and inhibit TNF-α, IFN-γ, FasR and JAK-STAT signaling pathways. Modulates the viral entry, fusion, replication, viral protein translation and virus budding of respiratory viruses	Phase I + II	[72,73,74,75]
Probiotics	Two capsules a day (one closed capsule to swallow and one open capsule mixed with maple syrup) from day 1 to 10 and one closed capsule to swallow from day 11 to 25, for maximum 25 days.	*Lactobacillus plantarum* DR7 suppress proinflammatory cytokines TNF-α, IFN-γ, enhancing anti-inflammatory cytokines IL-10, IL-4.	Not applicable	[71,75]
Quercetin	Patients will receive a daily dose of 400 mg of oral quercetin phytosomes	Dietary supplement: quercetin phytosomes	Phase 3	[74,75]

**Abbreviations:** TNF-α Tumor necrosis factor alpha; IFN-γ, Interferon gamma; IL, Interleukin; IKB-α, I-kappa-B-alpha; ROS, Reactive oxygen species; JAK, Janus kinase, NF-κB, Nuclear factor kappa B.

**Table 2:** List of plants against COVID-19 and their usefulness.

Plant/Family	Potential Bioactive Compound	Interventions	Ref.
*Curcuma Longa/*Zingiberaceae 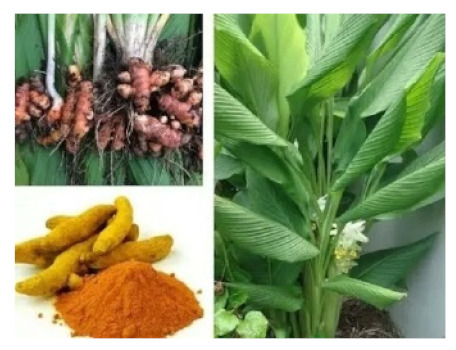	Curcumin 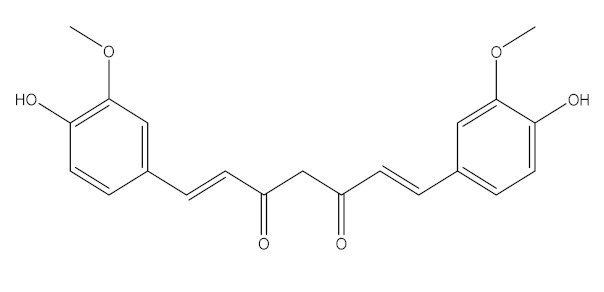	Increase the development of pro-inflammatory cytokines Rise in ACE2, accelerating the disease’s development	[21,97,100]
*Cinchona officinalis/*Rubiaceae 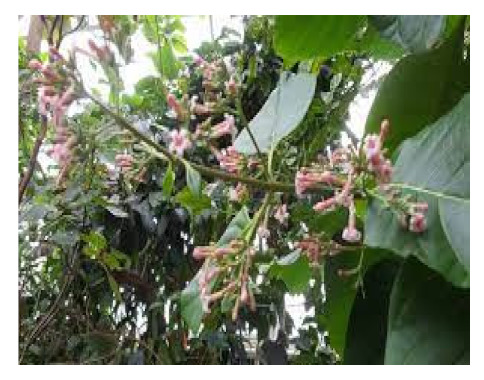	Quinine 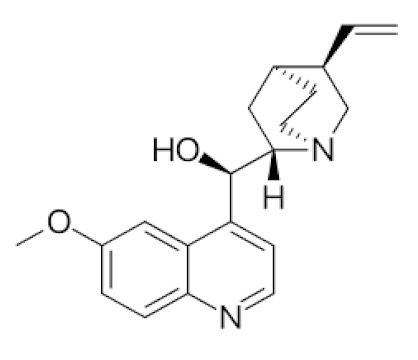	Chloroquine and Hydroxychloroquine are synthetic analogs of quinine, and both have promising role in reducing the SARSCoV-2 viral load in COVID-19 patients. LCCD widely used in treating COVID-19 patients.	[104,107,108]
*Allium sativum/*Alliaceae 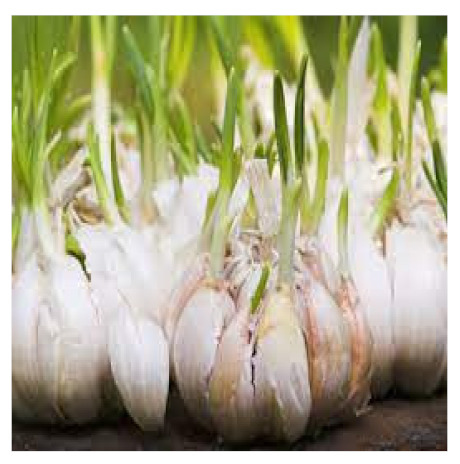	Allicin 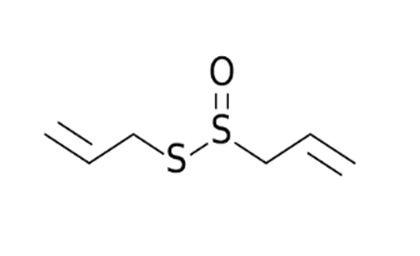	Reversal any of the signs and effects shown in these patients Restoration or recovery of the sensorial sensation that has been lost or diminished, Boosts the amount of Treg cells in the body Boosts the number of cytotoxic and helper T cells Lowers leptin, leptin receptor, and PPAR-I levels CD4+, Interleukin-2 receptor alpha chain, FoxP3+ regulatory T cells (Treg) cells from being inhibited Reduces the amount of IL-6 in the body Activate NK cells, Reduces TNF- and C-reactive protein levels	[109,110,147]
*Sambucus nigra/*Adoxaceae 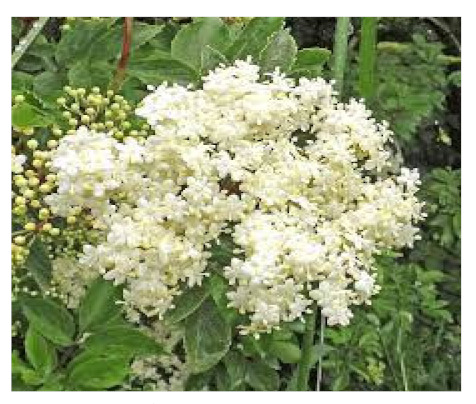	Quercetin 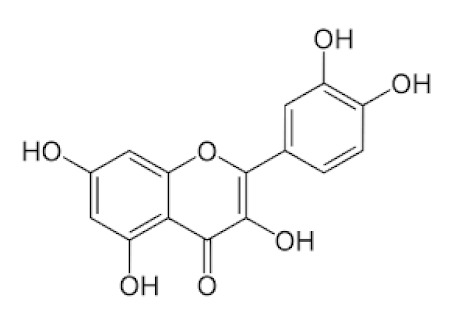	Reduces upper respiratory symptoms caused by viral infections and has potential immunological mechanisms of action	[116,117,118,148]
*Piper Nigrum L./*Piperaceae 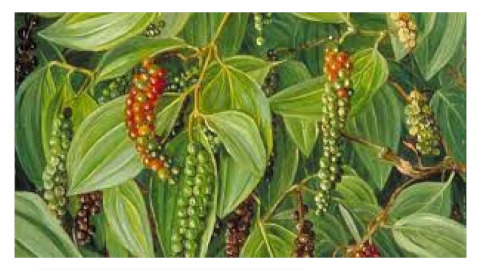	Piperine 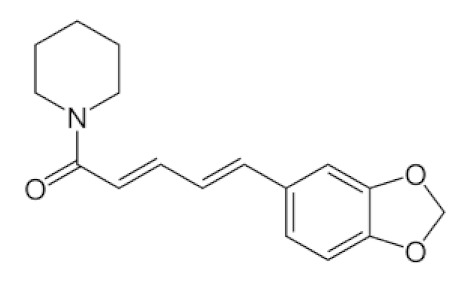	Reduces respiratory system infection have antiviral & antibacterial properties	[119,120,121]
*Vaccinium* berries/Ericaceae 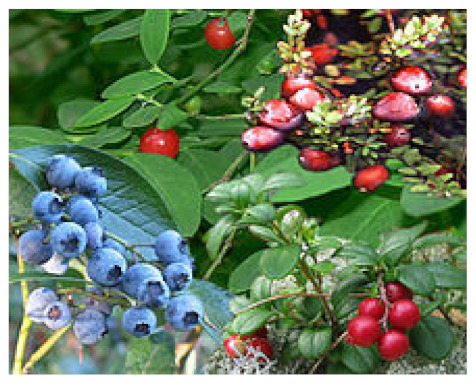	Resveratrol 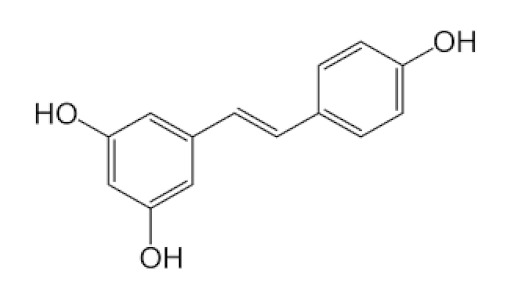	Blocks the key pathways involved in SARS-CoV-2 pathogenesis Controls RAS and expression of ACE, body’s immune activation Down-regulation of pro-inflammatory cytokines release.	[82,86,89,149,150]
*Withania somnifera/*Solanaceae 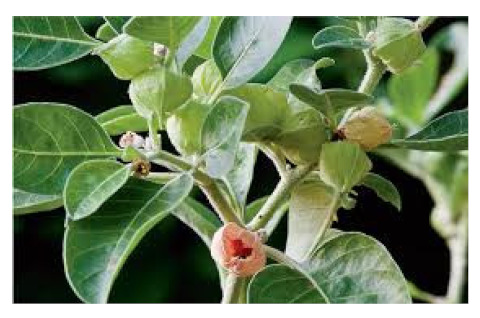	Withaferin A 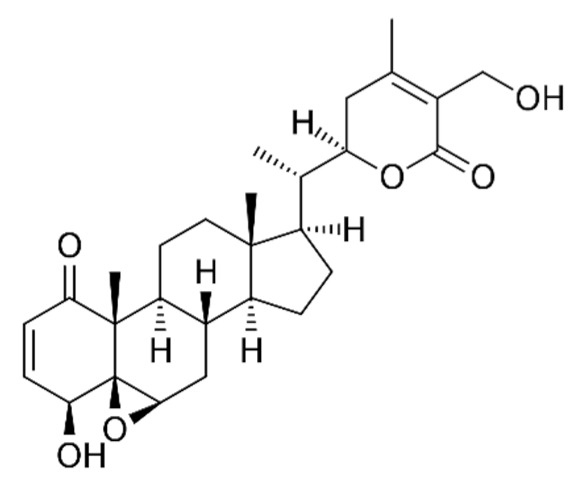	Lowering of pulmonary hypertension through decreasing inflammation, oxidative stress, epithelial dysfunction, respiratory cell apoptosis, and pulmonary respiratory system tolerance. Triggers the innate immune system and regulate the immune response of Th1 cells	[122,123,124,126,127]
*Ocimum sanctum/*Lamiaceae 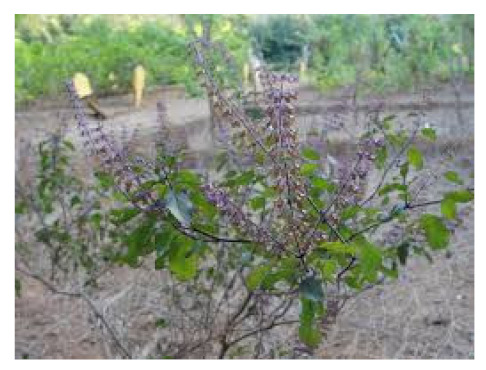	Eugenol 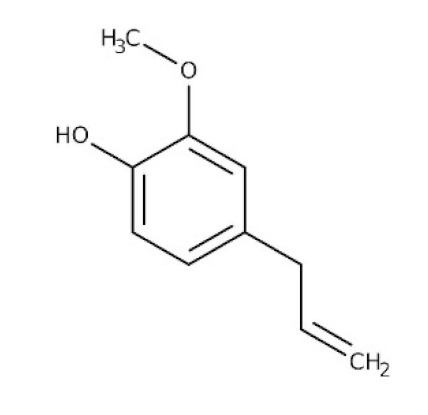	Cures lung infections and relieves pain, diarrhea, and coughing.	[87,130]
*Astragalus membranaceus/*Leguminosae 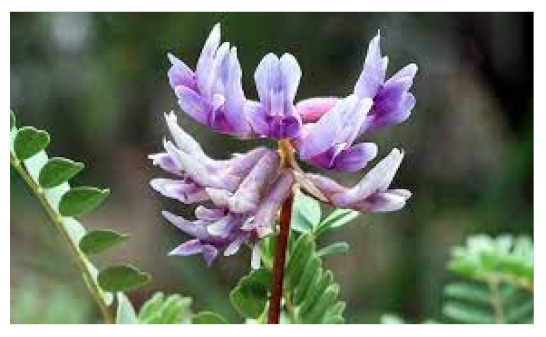	Astragalus polysaccharides 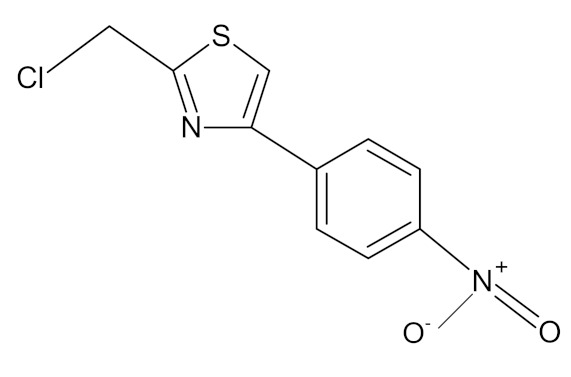	Immunomodulator and antiviral activity. Interferes with the entry process of virus by blocking the positive charge of the pathogen surface receptors, to prevent them from binding to HSPGs on the surface of host	[133]
*Nigella sativa/* *Ranunculaceae*	Thymoquinone 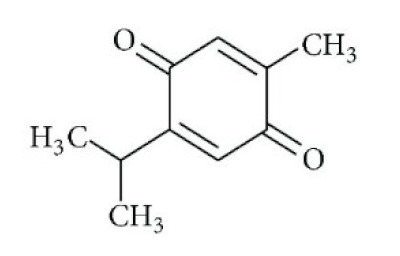	Antioxidant, anti-inflammatory, immunomodulatory, epigenetic modulation, antiviral activity, docking studies on anti-COVID-19 activity, antibacterial and anticoagulant effects for the treatment of COVID-19.	[134,135,136,151]
*Tinospora cordifolia/* *Menispermaceae*	Tinocordiside 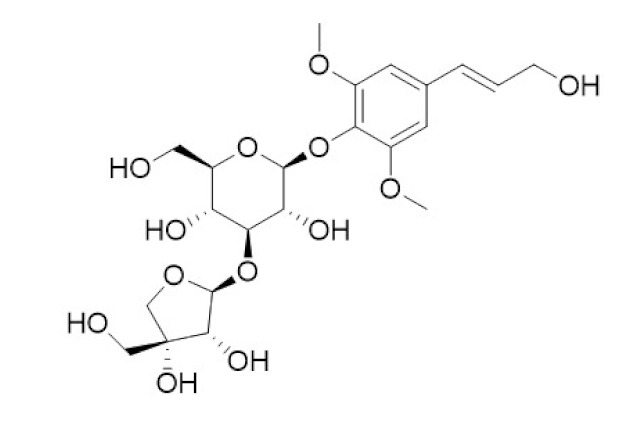	Highest binding affinity as compared to built-in ligand N3 for SARS-CoV-2 M^pro^ as per YASARA scoring.	[87,137,138,142,152]
*Glycyrrhiza glabra* L./ *Fabaceae*	Glycyrrhizin 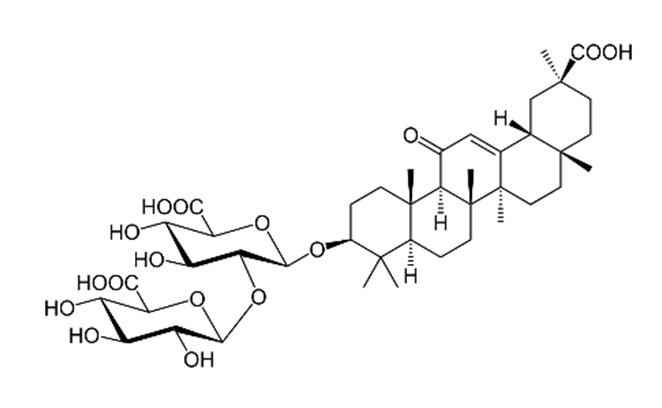	Interferes specifically with ACE2 Stopped FFM-1 and FFM-2 viruses from replicating and disrupted their biosorption and osmotic replication cycles.	[142,144,145]
*Zingiber officinalis/* Zingiberaceae	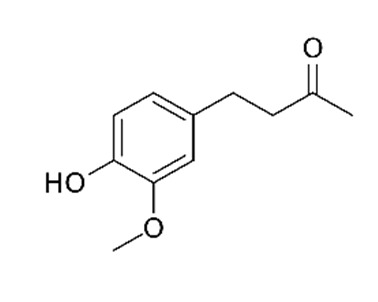 Zingerone 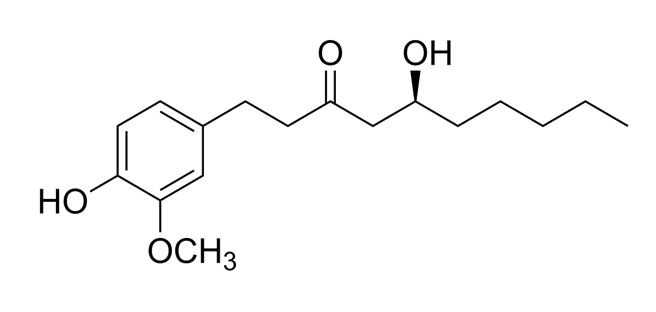 6-gingerol	Antimicrobial, antioxidant, and anti-inflammatory activities. Inhibits human respiratory syncytial virus	[146,153]

Legend: ACE2, Angiotensin-converting enzyme; SARS-CoV-2, severe acute respiratory syndrome coronavirus 2; LCCD, Lung cleansing and detoxifying decoction; CD4+, cluster of differentiation 4; FoxP3+, Forkhead box P3 protein; HSPGs, heparan sulfate proteoglycan.
